# University of the State of New York

**Published:** 1891-01

**Authors:** 


### UNIVERSITY OF THE STATE OF NEW YORK — EXAMINATIONS DEPARTMENT.

We publish the following instructions from the State Board of Regents for the information of all concerned:

*Endorsement of Medical Diplomas and Licenses Under the Laws of N. Y., 1887, Chap. 647, as Amended by the Laws of N. Y., 1890, Chap. 500.*

Candidates for endorsed diplomas or licenses should send to

Examinations Department, U. S., N. Y.: Albany, N. Y.: the affidavit, questions, and certificates of moral character on the enclosed forms; credentials of preliminary education; the local fee of ten dollars, and the medical diploma or license on which the endorsement is asked. The certificates of moral character should be from physicians legally authorized to practice medicine. The credentials of preliminary education should show that the candidate has passed the required Regents' examinations; or, if equivalents are offered, they should not cover less than one full year in a college, or three years in an institution of academic grade. The medical credentials will be presented for consideration to the State Board of Medical Examiners, selected by the candidate, and results will be returned as soon as possible.

RALPH W. THOMAS,
*Chief Examiner.*

Albany, N. Y., ............ 189

UNIVERSITY OF THE STATE OF NEW YORK — EXAMINATIONS DEPARTMENT.

This certifies that I have been personally acquainted with Dr. .................. for ...... years; that I know him to be of good moral character, and entirely worthy of confidence; and I hereby recommend him to the Regents of the University as entirely worthy to be licensed to practice medicine in the State of New York, pursuant to Chap. 647, Laws of N. Y., 1887, as amended by Chap. 500, Laws of N. Y., 1890.

..................................

Graduate of ........................

In the year..........................

Present residence ......................

QUESTIONS AND AFFIDAVIT.

1. What was your age when you began the study of medicine?

2. How many years have you spent in medical study with a preceptor?

3. How many regular courses of medical lectures have you attended, and at what medical institution or institutions?

4. Of what incorporated medical school or college are you a graduate; or, in what state or country did you receive a license to practice medicine?

5. Did this diploma or license entitle you to practice medicine in the state or country in which it was conferred?

6. Are you the identical person named in such credential?

7. Are you subject to any of the disqualifications for the study of medicine laid down in Laws of N. Y., 1887, Chap. 647, Sec. 6?

8. Have you ever practiced medicine? If so, where, and how long?

9. To what post-office address do you wish the endorsed credential sent?

Sign your name here . . . . . . . . . . . . . . . . . . . . . . . . . . . . .

STATE OF NEW YORK } ss.

County of . . . . . . . . . }

. . . . . . . . . . . . . . . . . . ., being duly sworn, says that h. . name is . . . . . . . . . . . . . . . . . . . ; that . . . was born in . . . . . . . . . . . . . . . ., on the . . . . day of . . . . . . . . . ., in the year . . . . ; that . . . . now resides at No. . . . . . . . . . . . . . Street, in the City of . . . . . . . . . ; and intends to practice medicine in the County of . . . . . . . . . ; that . . . . has written the answers to the questions hereto attached as such answers now appear, and that the facts set forth in such answers are true. And affiant further says that before receiving said . . . . . . . ., upon which . . . . desires an endorsement, . . . . . . fully and substantially complied with the requisites as to attendance, terms and amount of study and examinations required by the laws of the State and the charter and regulations of said . . . . . . . . . . . . . ., as preliminary and necessary to the conferment thereof. Affiant further says that no money was paid by . . . . for said . . . . . . . . . . . . . . ., except the regular fees paid by all applicants therefor; that no fraud, misrepresentation, or mistake in any material regard was employed by any one, or occurred in order that said . . . . . . . . . . . . . . should be conferred on affiant.

Sworn to before me }
this . . . day of . . . . . ., 189 } . . . . . . . . . . . . . . . . . . . . .

